# Variation in Plant–Pollinator Network Structure along the Elevational Gradient of the San Francisco Peaks, Arizona

**DOI:** 10.3390/insects12121060

**Published:** 2021-11-26

**Authors:** Paige R. Chesshire, Lindsie M. McCabe, Neil S. Cobb

**Affiliations:** 1 Department of Biological Sciences, Northern Arizona University, Flagstaff, AZ 86011, USA; 2 Biodiversity Outreach Network (BON), Mesa, AZ 86011, USA; neilscobb@gmail.com; 3 USDA-ARS Pollinating Insects Research Unit, Logan, UT 84341, USA; lindsie.mccabe@gmail.com

**Keywords:** pollination networks, plant–pollinator interactions, bees, flies, butterflies, pollinators, elevational gradient

## Abstract

**Simple Summary:**

Comparisons of plant and insect pollinator networks along elevational gradients can help predict future impacts of changing climate on pollinator distribution on local scales. We compare the pollination network structure along the altitudinal gradient of the San Francisco Peaks in Arizona. We evaluate shifts in network connectance, nestedness, modularity, and overall generalization with increased elevation. We conclude that plant–pollinator networks become more nested and generalized with elevation and identify the insect pollinator species most critical for network stability at the higher elevation pollination community. The variation in plant–pollinator network structure at different elevation zones of the San Francisco Peaks helps unveil which local communities currently support the most stable systems in the face of climate change.

**Abstract:**

The structural patterns comprising bimodal pollination networks can help characterize plant–pollinator systems and the interactions that influence species distribution and diversity over time and space. We compare network organization of three plant–pollinator communities along the altitudinal gradient of the San Francisco Peaks in northern Arizona. We found that pollination networks become more nested, as well as exhibit lower overall network specialization, with increasing elevation. Greater weight of generalist pollinators at higher elevations of the San Francisco Peaks may result in plant–pollinator communities less vulnerable to future species loss due to changing climate or shifts in species distribution. We uncover the critical, more generalized pollinator species likely responsible for higher nestedness and stability at the higher elevation environment. The generalist species most important for network stability may be of the greatest interest for conservation efforts; preservation of the most important links in plant–pollinator networks may help secure the more specialized pollinators and maintain species redundancy in the face of ecological change, such as changing climate.

## 1. Introduction

Insects, especially bees, are critical for pollination services worldwide [1,2,3]. Many fly species replace bees as the dominant pollinators at high elevation communities due to their ability to maintain functionality in cooler conditions [3,4,5]. Hoverflies (Diptera: Syrphidae) are especially important dipteran pollinators who feed almost exclusively on the nectar and pollen of many wild plants and important food crops [6]. However, increased global warming, climate variability, and land use change is leading to higher temperatures, habitat loss, and resource loss for insect pollinators, already affecting species distribution and diversity across time and space [7,8,9,10,11]. With temperatures predicted to warm ~3 °C over the next 80 years [12], there may be increased instances of phenological asynchrony, or a disruption in the overlap of pollinator foraging time and host plants’ flowering period [13,14]. Most plant species in temperate environments rely on air temperature as a trigger for flowering, and even a six-day premature blooming period has been shown to disrupt pollinator–plant associations and negatively impact the fitness of solitary bee species [15]. Additionally, a rise in ambient temperature may cause insect pollinators to shift upward in elevation or latitude to follow the climate with which they are adapted, potentially leaving pollinators with a new suite of host plants in their new range [13,14,16,17,18,19]. Insect pollinator species range from being dietary specialists to extreme generalists; obligate specialists depend on one or a few plant species while extreme generalists may use numerous floral resources available in their community [20,21,22]. Previous research has shown that pollinator specialization can vary along productivity gradients [22,23], and while some bee, fly, and butterfly species may be able to act as opportunistic generalists in the face of reduced host partner availability, others may be more sensitive to resource loss. The structural variability of plant–pollinator network interactions along environmental gradients can help predict how future shifts in temperature, extreme climatic events, or changes in species composition may impact pollinator community robustness.

Elevation gradients offer unique opportunities to study pollination network properties across different habitats and temperatures within a small geographic area, and these gradients represent suitable proxies for climate change by essentially replacing time with space [3,4,16,24,25]. In this study, we compare the structure of pollination networks at three elevation zones of the San Francisco Peaks in northern Arizona to determine which plant–pollinator communities in this local montane environment are less vulnerable to changing climate [4,26,27,28,29]. Namely, we analyzed network connectance, nestedness, modularity, specialization, and pollinator robustness. Network connectance reveals the proportion of observed plant–pollinator interactions out of those that have the potential to occur [26,30]. Networks consisting of more generalized species with a wider diet breadth are typically more connected due to an increased number of observed linkages with plants [23,31,32]. Nestedness describes the degree to which specialist species interact with partners who also associate with the highly generalized species [13,16,26,31,33]. More nested networks can offer built in redundancy and buffer the plant–pollinator system against species loss [34]. Network modularity characterizes the extent to which clusters of interacting species form tightly linked compartments that share very few interactions with other distinct compartments [35,36], and network specialization describes the level of overall species selectiveness within the plant–pollinator system [23,37]. Finally, robustness describes a network’s susceptibility to community collapse based on species extinction. Specifically, we measure overall pollinator robustness to plant species loss [38].

Previous studies have shown increases in connectance, nestedness, and species generalization, as well as decreased modularity, with increasing altitude [4,23,31], often driven by reduced partner availability because of decreased species richness. Increased elevation in temperate zones is typically associated with colder temperatures, reduced land area, shorter growing seasons, or unpredictable reproductive success of flowering plants, which can impose harsher conditions for flora and fauna [16,36,39]. Conversely, warmer conditions at lower altitudes may allow for longer flowering times and potentially increased reproductive success, positively affecting mass flowering of plant species [16]. Since different pollinator taxa groups have varying responses to weather and climate, some high elevation insect communities may experience increased environmental filtering and/or decreased species richness [16,23,24,28,36,39]. Larger-bodied, cold-adapted pollinators such as bumble bees and non-syrphid flies may dominate higher elevations of temperate regions whereas smaller-bodied, solitary bees drop out of the species pool. [3,40,41]. We have three predictions: (1) Plant–pollinator networks will become more connected and nested with increased elevation zone on the San Francisco Peaks; with fewer available host plant species, insect pollinators may be required to act as opportunistic generalists and share more plant resources [28]. (2) Network specialization and modularity will decrease with elevation zone. Modularity tends to increase with increasing link specificity [23], and therefore, lower overall selectiveness of plant partners at higher elevations may also lead to networks with fewer distinct modules. (3) The more generalized pollinators at the high elevation network will be the most critical species for plant–pollinator community stability and robustness. Dietary generalist pollinators often drive the more nested pollination networks [42], and with a wider flexibility in interaction partners, they may offer greater species redundancy in the event of host plant loss [20,28,43,44,45]. Identifying which pollinator species and interactions on the San Francisco Peaks currently support the pollination communities most robust to species loss may reveal which pollinator taxa contribute most to facilitating network success and structure [46]. Regardless of current population status, any event that could lead to the loss of these critical generalist species may lead to a cascading decline in diversity and/or community collapse [13,26,43,47].

## 2. Materials and Methods

### 2.1. Study Location and Sampling Methods

Located in northern central Arizona, ~20 miles north of Flagstaff (35.341031 N, −111.683217 W), the San Francisco Peaks is an especially important elevation gradient with high insect pollinator diversity, including at least ~360 native bee species [48]. The San Francisco Peaks make up one of two dozen “sky islands” in the southwest United States, or isolated mountain tops, that serve as biodiversity hotspots due to the multiple habitats present along the altitudinal gradient [49,50]. The San Francisco Peaks is characterized by the C. Hart Merriam elevational gradient that spans seven life zones (vegetation zones) and ranges from low-elevation desert ecosystems to high-elevation forest types (from 785 to 3850 m) [51]. Collection sites were established at three life zones at three distinct elevations: ponderosa pine (~2200–2500 m), mixed conifer (~2550–2700 m), and spruce fir (~2750–3100 m). We established six sites at each life zone (Supplemental Table S1), all at least 1 km apart, and each site was further divided into three 60 m × 2 m transects. All bees, flies, butterflies, and moths present on flowers were captured using a combination of sweep nets and modified hand vacuums [26] and the host plant was recorded. Pollinators were collected along each transect for 30 min by a single person. We recorded the number of plants, flowers, and buds for each species along a transect. A sampling period consisted of three consecutive days, with each day dedicated to sampling all six sites of a life zone during peak foraging time (9 a.m.–3 p.m.). If severe thunderstorms prevented sampling on any given day, we conducted additional sampling on a 4th or 5th consecutive day. The dates of each sampling period used for analyses are noted in Table 1. The order in which we visited the sites in each sampling period was randomized to capture pollinators with different foraging times. All insects were brought back to the lab for curation and identification. We had four distinct sampling periods throughout July-–August in 2017 and five distinct sampling periods during July–August in 2018. Data were combined across years but kept separate for each life zone.

### 2.2. Species Identification

All insects were initially identified in the Northern Arizona University (NAU) pollinator lab and digitally catalogued in the Symbiota Collection of Arthropods Network (SCAN) online database. Bees were identified using published identification guides (see methods from McCabe et al. [48]). Classification for species *Melissodes* and *Andrena* followed LaBerge [52,53,54] with modifications from Wright’s work on *Melissodes* [55,56]. All other bee species identifications followed the classification of Michener [57]. Identifications were confirmed by Jason Gibbs, University of Manitoba (*Lasioglossum*), Karen Wright, Texas A&M University *(Melissodes*), Harold Ikerd, USDA-ARS (Andrenidae), and Terry Griswold, USDA-ARS. Numerous fly specimens were identified to tribe, genus, or species by John Carr, MIT, through examination of images photographed with NAU’s macro-imaging system. Additionally, we used BugGuide.org to navigate through identification pages for individual taxa as well as uploaded macro-images of specimens to the “ID Request” page on the website. Furthermore, we used published identification guides, including Norrbom et al.’s “New genera, species and host plant records of Nearctic and Neotropical Tephritidae (Diptera)” and published Diptera textbooks, including Marshall’s “Flies: The natural history and diversity of Diptera”, to identify additional fly specimens [58,59,60]. Butterflies and moths were identified using K. Kaufman and J. Brock’s Field Guide to the Butterflies of North America [61] and identification pages for certain genera on BugGuide.org (e.g., *Scythris*). Insect specimens that could not be identified to species at this time were assigned a morphospecies designation, where specimens with similar morphological distinctions were grouped [48]. Each morphospecies was classified by the genus (and subgenus if determined) followed by a unique three-digit number. Most plants were identified to genus or species in the field and confirmed by the herbarium of NAU through examination of pressed field samples. Four additional plant species (*Lupinus argenteus*, *Gentiana affinis*, *Tragopogon dubius*, and *Lithospermum multiflorum*) were determined through research grade identifications on iNaturalist.

### 2.3. Comparisons of Plant and Pollinator Richness and Abundance across Life Zones

We first identified any shifts in plant and pollinator species richness across the three life zones surveyed on the San Francisco Peaks. We used Generalized Linear Models (GLMs), with life zone as the explanatory variable, to compare the average species richness of both plants and pollinators as the response variables. For pollinators, data did not follow a normal distribution, so we used a Poisson family distribution for this model. Plant species richness data followed a normal distribution, so we used a Gaussian family distribution. All analyses were run using R statistical software version 4.0.2. Additionally, we computed estimated marginal means (EMMs) using the *emmeans* package version 1.6.3 [62] to compare pair-wise differences in mean species richness of plants and/or pollinators between life zones. Due to inclement weather, two sites at the mixed conifer life zone could not be sampled on the week of 7/25/17 and one site at the ponderosa pine life zone could not be sampled on the week of 7/30/18 (Table 1). We also report total species richness observed at each life zone over the course of the study. We also identify any shifts in plant and pollinator abundance across the three life zones of our study area. We again used GLMs, with life zone as the explanatory variable, to compare the average abundance of plants and pollinators as response variables. Abundance data for both flowers and insects were non-normally distributed. For insects, we used a Poisson distribution. For flower abundance, the variance was greater than the mean, and therefore, we used the more appropriate quasi-Poisson error distribution. We computed EMMs to compare mean plant and/or pollinator abundance between life zone pairs. We also report total abundance of pollinator/flower individuals sampled at each life zone over the course of the study.

### 2.4. Network Indices Calculations

To test our first two predictions, we calculated five indices to describe network structure and/or the degree of generalization at each life zone using the “bipartite” package version 2.16 [63] in R. *Weighted connectance* (C) is the proportion of observed plant–pollinator links (*L*) out of those that have the potential to occur, ranging from 0 (no connectance) to 100 (perfectly connected). Weighted connectance uses Shannon’s diversity index to account for fluctuations in “link weights” that can arise when interactions have varying degrees of magnitude [64]. *Nestedness* describes the degree to which more specialized or poorly linked species interact with the species making up the more generalized subsets of the network [15]. Nestedness was expressed using the NODF index (nestedness based on overlap and decreasing fill) [4,23,26,65,66,67]. NODF is a revised nestedness measure based on overlap and decreasing fills in a matrix and ranges on a percentage scale of 0 (no nestedness) to 100 (perfectly nested) [26,27,65,68]. This metric is especially appropriate for bimodal interaction networks as it is standardized and not biased by network size [26]. *Modularity* (Q) measures the degree to which plant–pollinator species interactions are grouped into distinct modules (compartments) that show little interaction with other compartments [23]. More compartmentalized networks have values closer to 1 and are organized into groups where interactions are strong within but are sparse between. *Network specialization* (H_2_) describes the selectiveness of interaction networks and how observed linkages deviate from those expected based on species totals [37,69]. Values range from minimum specialization (0) to maximum specialization (1) [27]. *Robustness* (R) quantifies a network’s susceptibility to community collapse because of species extinction. We calculated overall pollinator robustness in the face of plant extinction. Highly robust systems tend toward a value of 1, whereas values closer to zero correspond to networks that experience abrupt species loss in the event of one or two initial extinctions [37,42]. Networks were constructed at a site level (six per life zone) and quantitative values for all five indices of interest were calculated at each network. We used GLMs to compare network structure on the San Francisco Peaks, with life zone as the explanatory variable and each of the calculated network metrics as response variables. Connectance data were normally distributed, so we used a Gaussian family distribution. Data for the other four network metrics were non-normally distributed and we used a Poisson family distribution. We again computed EMMs to compare means between life zone pairs. We report overall values for connectance, NODF, modularity, network specialization, and robustness at each life zone by averaging those obtained at site-level network analyses.

### 2.5. Core Generalist Species Calculations

We determined core generalist pollinator species for all life zones using the formula Gc = k_i_ − k_mean_/O_k_′ [26,27], where k_i_ is the mean number of links for a given pollinator species, and k_mean_ and O_k_ are the mean and standard deviation among pollinators, respectively, for k. Gc values >1 indicate core generalists as they have a larger number of interactions relative to other species in the network [27]. Gc values <1 are species with a lower number of interactions in relation to other pollinator species and constitute the periphery of networks [27]. To calculate core generalists, data from all six sites were pooled for a single life zone, such that each life zone had one set of plant–pollinator interactions and plant/pollinator species observed. For each life zone, we calculated the percentage of total individuals made up by core generalist species and the percentage of unique plant–pollinator associations made up by core generalist species.

### 2.6. Generalist Contribution to Network Stability at a High Elevation Plant–Pollinator Community

Based on methods created by Alarcon et al. [70], all species in a network can be assigned a nested “rank”, measured as the position in the nestedness matrix, according to their generality [63]. Using this method, the most generalized species is ranked number 1, the most specialized species is at the bottom, and all other species are ordered appropriately in between [70]. We use the bipartite package [63] to calculate a nested rank for all pollinator species at the higher elevation community. Values closer to 1 mean that the species are more generalized, and higher values correspond to more specialized species in the network. We simulate extinction events by removing the three most generalized pollinator species from the networks as well as three more specialized pollinator species from the networks at all six spruce fir sites to investigate how nestedness may change when species with varying degrees of importance are lost from the pollination system. New nestedness values were averaged across all six sites after each pollinator was removed from the community.

## 3. Results

A total of 1703 insect specimens for 155 species were collected across our study area (Table 2). Flies were the most abundant insects sampled, making up 57% of the individuals collected. This pattern holds true when analyzed by individual life zone (flies make up 60%, 55%, and 58% of individuals at the ponderosa pine, mixed conifer, and spruce fir life zones, respectively). We encountered 98 flowering plant species during our study, but only 47 were observed interacting with pollinators. We report information on plant abundance, richness, and overlap across life zones using only the subset of species seen to interact with a pollinator, however we acknowledge that other resources at these life zones may have been visited by pollinators outside of our sampling times.

### 3.1. Pollinator and Plant Species Richness by Life Zone

**Figure 1:**
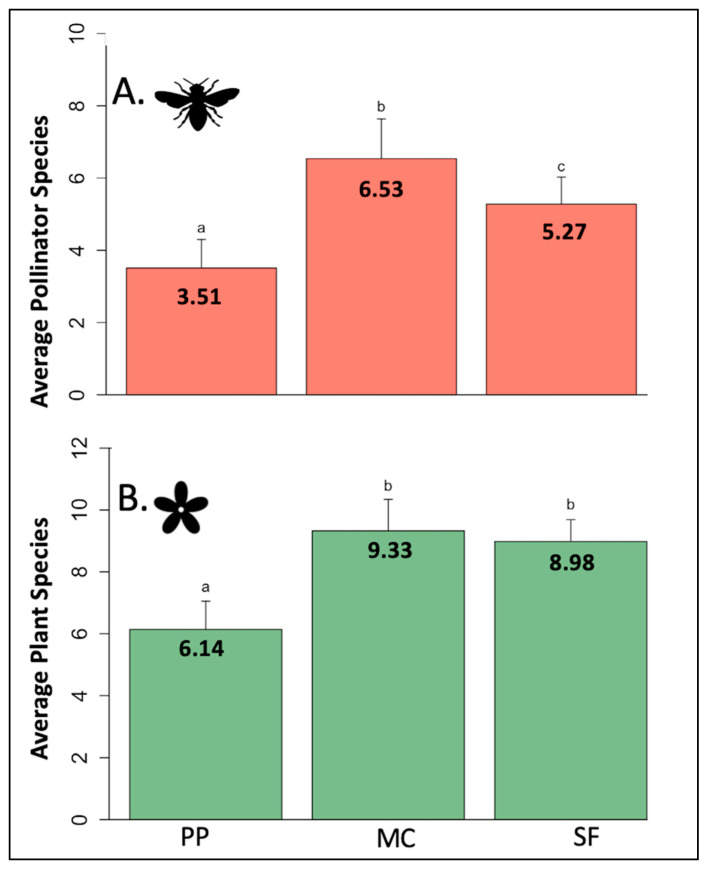
Average pollinator (**A**) and plant (**B**) species richness by life zone. PP = ponderosa pine ~2200–2500 m), MC = mixed conifer (~2550–2700 m), SF = spruce fir (~2750–3100 m). Averages were calculated using all 30-min sampling events, regardless of site number or sampling week. Letters denote significance.

Total pollinator species richness peaked at the middle life zone (mixed conifer: 102 species), whereas 73 and 72 species were collected at the lower and higher elevation zones, respectively. Average pollinator species richness was significantly different across all life zones (GLM: df = 2, F = 24.618, *p* < 0.001), with that at the mixed conifer life zone being significantly higher than both the ponderosa pine life zone (EMM: z = −6.823, *p* < 0.001) and the spruce fir life zone (EMM: z = 2.667, *p* = 0.021) (Figure 1A).

Total host plant species richness showed a similar pattern as pollinator species richness; the mixed conifer life zone had 32 host plant species, compared to 27 and 20 plant species collected at the lower and higher zones, respectively. Average plant species richness was significantly different across life zones (GLM: df = 2, F = 14.862, *p* < 0.001), where the ponderosa pine life zone had a significantly lower average number of plant species than the mixed conifer life zone (EMM: z = −4.979, *p* < 0.001), however the decrease between the mixed conifer and spruce fir life zones was not statistically significant (EMM: z = 0.539, *p* = 0.852) (Figure 1B).

### 3.2. Pollinator and Flower Abundance by Life Zone

**Figure 2:**
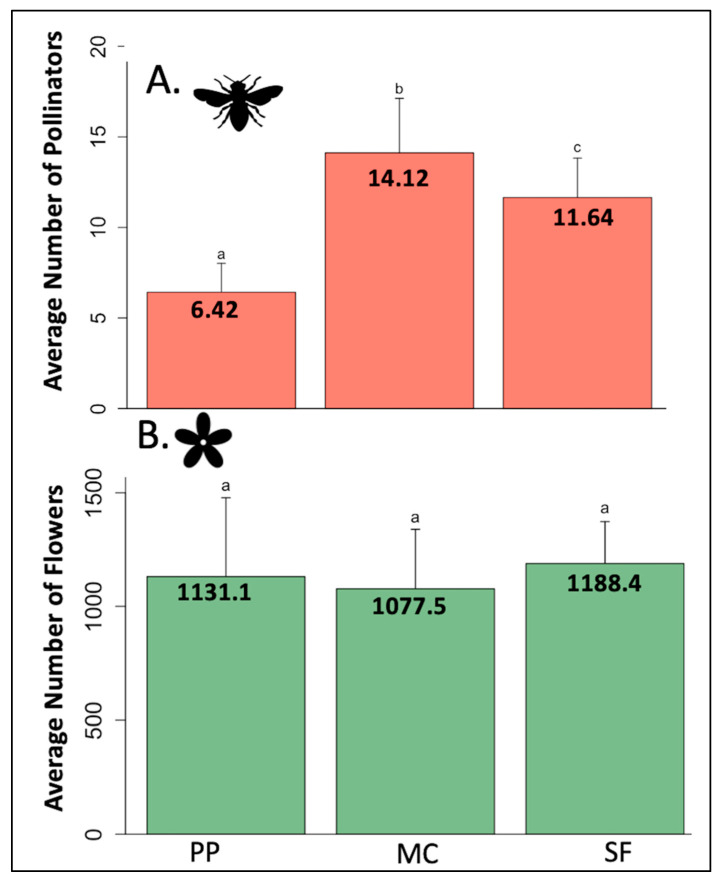
Average pollinator (**A**) and flower (**B**) abundance by life zone. PP = ponderosa pine (~2200–2500 m), MC = mixed conifer (~2550–2700 m), SF = spruce fir (~2750–3100 m). Averages were calculated using all distinct 30-min sampling periods, regardless of site number or sampling week. Letters denote significance.

The greatest number of insect individuals was also collected at the mixed conifer life zone (734 individuals) compared to 340 and 629 at ponderosa pine and spruce fir life zones, respectively. Although the higher elevation life zone had a greater number of pollinator individuals than the lower elevation ponderosa pine, this could be due to the high count of *Fannia* sp. (morphospecies *Fannia 001*) (212 individuals) present in this habitat. Average pollinator abundance shows a similar hump-shaped pattern (GLM: df = 2, F = 81.097, *p* < 0.001), where that of the mixed conifer life zone is significantly higher than both ponderosa pine (EMM: z = −12.021, *p* < 0.001) and spruce fir (EMM: z = 3.536, *p* = 0.001) life zones (Figure 2A).

Total flower abundance was similar across all life zones (ponderosa pine = ~64 k, mixed conifer = ~62 k, and spruce fir = ~67 k) and average flower abundance was not significantly different across life zones (GLM: df = 2, F = 0.158, *p* = 0.854) (Figure 2B).

### 3.3. Network Structure by Life Zone

**Figure 3:**
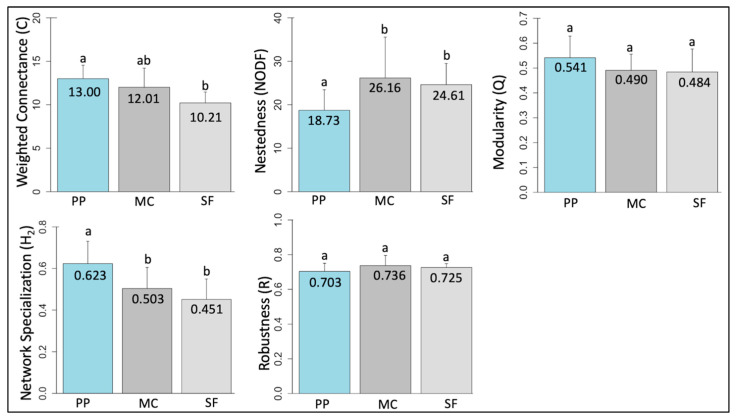
Quantitative values of each network property reported by life zone. Values were obtained from site-level network analyses and averaged across life zones. PP = ponderosa pine (~2200–2500 m), MC = mixed conifer (~2550–2700 m), SF = spruce fir (~2750–3100 m). Letters denote significance.

**Figure 4:**
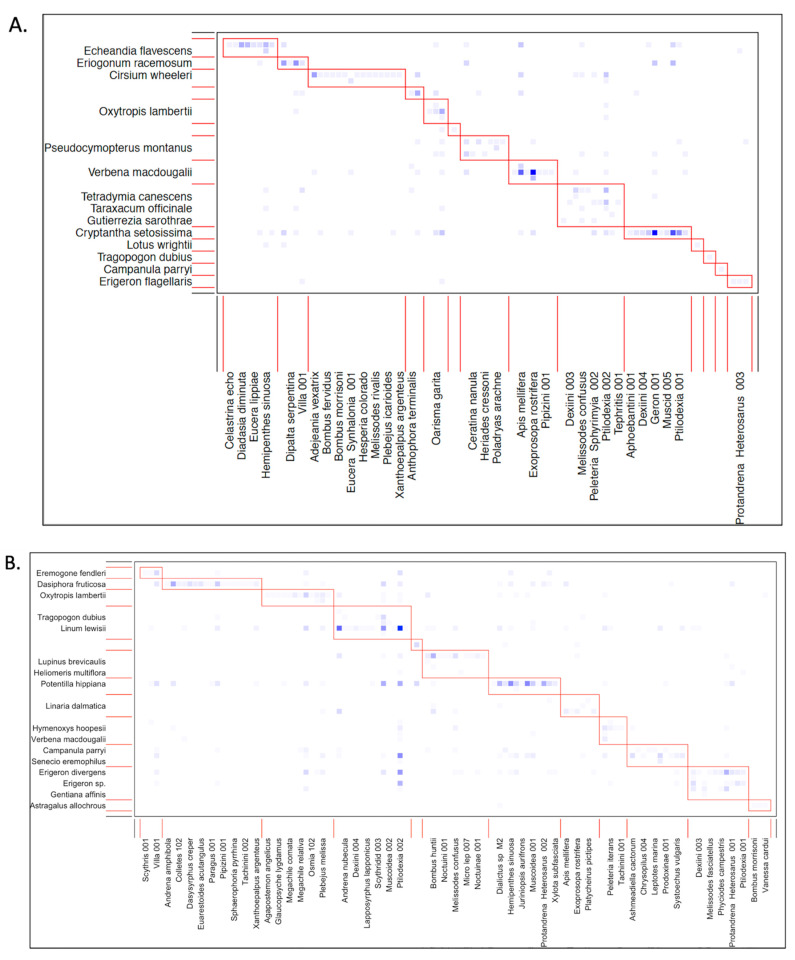

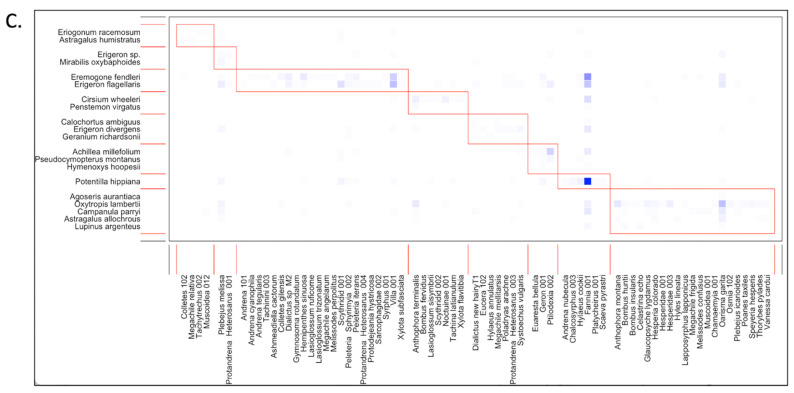
Modularity plots for plant–pollinator community interactions between plant and pollinator species at each life zone. Plant species are listed along the *y*-axis and pollinator species are listed along the *x*-axis. The plots show interaction data combined across all sites for a life zone, although analyses were conducted on a per-site basis. (**A**) = ponderosa pine (~2200–2500 m), (**B**) = mixed conifer (~2550–2700 m), (**C**) = spruce fir (~2750–3100 m). Distinct boxes around subsets of species interactions represent compartments that have many links within but few between. Darker blue squares represent higher numbers of associations documented for that pair.

The largest plant–pollinator network occurred at the mixed conifer life zone; not only did this life zone have the highest total number of plant and pollinator species, but also the greatest number of unique plant–pollinator associations (Table 3). Contrary to our predictions, there was no significant difference in network connectance across life zones (GLM: df = 2, F = 2.608, *p* = 0.107) (Figure 3). However, as expected, nestedness varied with life zone elevation (GLM: df = 2, F = 4.108, *p* = 0.017), although the difference between the two higher zones mixed conifer and spruce fir was not statistically significant (EMM: z = 0.532, *p* = 0.856). Further, there was no significant difference in network modularity (Q) with increased life zone elevation (GLM: df = 2, F = 1.150, *p* = 0.317) (Figure 4). As expected, there was a significant decrease in network specialization (H_2_) along the gradient (GLM: df = 2, F = 8.739, *p* < 0.001), where the spruce fir life zone had the lowest degree of selectiveness (H_2_ = 0.451), although the difference between the mixed conifer and spruce fir life zones was not statistically significant (EMM: z = 1.319, *p* = 0.384). Interestingly, overall pollinator robustness (R) to plant extinctions was not significantly different along the gradient (GLM: df = 2, F = 0.241, *p* = 0.786). Graphs of all species and interacting links observed across a life zone are reported in Supplemental Figures S1 and S2.

### 3.4. Core Generalist Pollinators by Life Zone

The spruce fir life zone had the fewest number of “core generalist species” with Gc values >1.0, yet the numerical values of the Gc values are, on average, much higher than those at lower elevation life zones (Table 4). The ponderosa pine life zone may have produced a greater number of species with Gc values >1.0 due to the higher number of singleton pollinator species in that community. Specifically, 25% of interactions at the ponderosa pine life zone were comprised by singletons compared to 18% at the spruce fir life zone. Networks with more singleton species will show a lower average number of plant partners per pollinator, such that any pollinator that is not a singleton has a better chance of producing a Gc value greater than 1.0. For example, the fly species *Phthiria* sp. (morphospecies *Phthiria 003*) only interacted with three plant species at ponderosa pine, yet it received a Gc value > 1.0.

### 3.5. Generalist Importance at the High Elevation Plant–Pollinator Community

Flies comprise most interactions at the higher elevation community, and the importance of butterflies and moths also increases relative to lower life zones (31% of interactions at spruce fir, compared to 16% and 22% for ponderosa pine and mixed conifer, respectively). The top three generalist species at the spruce fir life zone, two butterfly species (*Oarisma garita* and *Plebjeus melissa*) and one fly species (*Fannia 001*), are responsible for 18% of interactions in this network. These three pollinator species also have the highest nested ranks in the network (Table 5). When statistically simulating the removal of these top three generalists, network nestedness decreases by 36.77% (Table 5). Conversely, if experimentally removing three more specialized pollinator species at the spruce fir life zone, there is little to no decrease in nestedness, and in some situations, nestedness shows a slight percent increase (Table 6).

## 4. Discussion

Our results confirm variations in plant–pollinator network structure along the elevational gradient of the San Francisco Peaks. Total species richness of both plants and pollinators loosely follows the unimodal, hump-shaped pattern often observed along elevational gradients [39,71], with the highest species richness present at the mixed conifer life zone. This may be attributed to an overlap in species ranges at the mixed conifer life zone; for example, 74% of lower-elevation pollinator species and 66% of higher-elevation pollinator species are also present at this life zone (Supplemental Table S2). Similarly, 65% of the spruce fir life zone host plants and 63% of the ponderosa pine life zone host plants are also host species at the mixed conifer life zone (Supplemental Table S3). The subsequent decrease in species richness from the mixed conifer life zone to the higher zone spruce fir is consistent with patterns observed in other studies of plant and pollinator richness along elevational gradients [16,23,28]. A combination of fluctuating climatic factors at higher elevations that determine the productivity of a plant–pollinator community, including precipitation, temperature, and solar radiation, may drive the taxonomic groups and/or number of individuals able to exist at certain geographic zones [39,72]. One recent study [16] found significant decreases of plant and insect species at higher elevations of Jonaskop Mountain, South Africa, and similarly, research in the Andes of Mendoza, Argentina [31], showed that the number of pollinator species decreased by over 33% from a lower to higher elevation zone. Further, decreasing temperatures at the higher elevations of the Mexical scrublands supported declines in bee species richness, with mean annual temperature as the best predictor of declines [1]. Cooler temperatures can affect the physiological and foraging capabilities of insects, which can lead to overall environmental filtering on bee or other insect communities along altitudinal gradients [1,16,72,73]. Average pollinator species richness also followed a significant hump-shaped pattern, but average plant species richness was not significantly different between the mixed conifer and spruce fir life zones. While both average and total pollinator abundance followed a hump-shaped pattern, average and total flower abundance was equal across life zones. This suggests that (a) there is a greater proportion of flowers per species at higher elevations or (b) the extremely high number of one or two dominant plant species at the spruce fir life zone, including *Oxytropis lambertii* and *Eremogone fendleri*, may be inflating the number of flowers counted at the higher elevation life zone.

When pollinator species communities vary locally across the range of plant species, such as along environmental gradients, there may be intraspecific variation within the plants that reflect adaptive shifts to different pollinator presence [74,75]. Previous research has shown that plant communities may vary along an elevational gradient; because of limiting climatic factors at higher altitudes, such as decreased temperature and decreasing atmospheric pressure [72,75], there may be intraspecific morphological changes within plant species, such as decreased overall size, longer growing periods, shorter maximum height of individuals, and larger seed mass [16,75,76]. Local selection of beneficial traits for plant individuals at different climatic zones may assist with survival in harsher habitats [75]. Additionally, some plants produce larger flowers at higher elevations, where larger-bodied insects, such as bumblebees and flies in the family *Bombyliidae*, often dominate high altitudes [16]; an increase in flower size may be more attractive to these specific pollinators and increase chance of pollination [74]. Besides morphological variation, plants may become more specialized at lower elevations, which could be occurring within our study area. For example, *Oxytropis lambertii* (purple locoweed) was highly abundant at all elevations on the San Francisco Peaks. At the high elevation spruce fir, *O. lambertii* interacted with 33% of pollinator species and 14% of individuals. While it had a similar importance at the mixed conifer life zone, *O. lambertii* only interacted with 2% of individuals and 5% of species at the ponderosa pine life zone. This suggests that this plant becomes more generalized with increased elevation.

Some network features showed variation as predicted along the gradient. For example, there was a significant decrease in network specialization (H_2_) with elevation. This supports overall lower plant selectiveness at the higher elevation life zone of the San Francisco Peaks. It is possible that the more generalized pollinator species at the spruce fir life zone have greater weight within the network and help to secure the poorly linked (more selective) species across the community [7,13,27,77]. Further, nestedness was significantly lower in the lowest life zone. These patterns may be driven by a decrease in total species richness of flowering plant partners at the higher elevation life zone, spruce fir; with fewer host-plant options, insect pollinators may choose to interact with the more stable plant species in a community. These findings are consistent with those of recent studies of plant–pollinator network structure along elevational gradients; for example, research in the Canary Islands [23] found a decrease in network specialization at higher elevations of the El Teide stratovolcano, likely explained by reduced partner availability leading to a wider niche breadth. Similarly, research in Germany [28] found that higher elevation plant–pollinator communities of the northern limestone Alps, especially when confronted with an experimental treatment of delayed snowmelt, showed greater generalization than lower elevations.

Contrary to our expectations, however, connectance did not show significant differences with life zone. This could be a product of small sample sizes when running analyses at a site level. For example, at the spruce fir life zone, when data are kept separate across sites, some pollinator species appeared to be singletons who only interact with one plant, including *Bombus huntii*. However, when data are pooled, many of these pollinators are actually more generalized as they interact with multiple plant species across the life zone. Thus, the contribution of what appear to be, but are not actually, singleton interactions at individual spruce fir sites may be depressing connectance values of this higher elevation life zone. This underscores the importance of adequate sample sizes to accurately identify the plant–pollinator interactions comprising a pollination system. Additionally, although we expected modularity to be higher at the lower elevation life zone ponderosa pine due to greater specialization, there were no significant differences in modularity across life zone. It is possible that high modularity did not emerge in any of our pollination systems due to small network sizes when analyzed at a site level. It has been shown that modularity may be less pronounced or absent in smaller networks (<50 species), whereas it is common in larger networks (>150 species) [26,78]. At individual sites, even the most species rich network only had a total of 56 plant and pollinator species sampled throughout our study period, which may make it difficult for patterns of modularity to emerge in our small pollination networks. Other studies of network structure along elevational gradients have also identified patterns that differ from what we expected. Research in Chile [36] found that modularity was actually conserved along an elevational gradient in the Andes, and that nestedness decreased, even though there was a simultaneous increase in connectance. Similarly, while studies on Mt. Olympus, Greece [4] did show increased nestedness in bumblebee-plant networks at high elevation communities, there was no correlation between network specialization and elevation. This highlights how network indices are not always predictably correlated and that results may vary depending on the pollinator taxa groups included in the network analyses.

Typically, networks with higher nestedness and generalization show greater robustness when it comes to ecological disturbances such as changing climate or decreasing available habitat [35,43]. Although the robustness coefficient (R) was not significantly different along the altitudinal gradient, of interest at the spruce fir life zone are the highly interactive generalist species identified, including *Plebejus melissa* and *Oarisma garita*, as they may facilitate the construction of nested subsets and higher overall generalization. It is important to acknowledge the relationship between species Gc values and abundance at all life zones; recent studies suggest that abundance may influence species persistence in a habitat and that this bias is often ignored when identifying generalist species [32]. Specifically, the more abundant species are likely to be sampled more frequently [32], potentially skewing the results of which pollinators have the widest diet breadth. At all three life zones in our study, species abundance showed significant positive correlations with calculated Gc values, such that the species considered “core generalists” are typically more abundant in the community. This may have played a role in the calculation of generalist pollinator species used in our analyses (Supplemental Figure S2).

The simulations of pollinator species extinction at the spruce fir life zone showed that a loss of the three most generalized pollinators resulted in lower network nestedness. Although these pollinators may currently be abundant and stable in these pollination communities, increasing temperatures and greater instances of extreme climatic events could put their population health at risk. For example, increased drought frequency and intensity is predicted across regions worldwide, which can severely lower the amount of available floral resources for even the more stable, generalized pollinators [79]. This has already occurred in places that affect the area represented in this study; in June 2021, a 6-day period of record-breaking heat in the southwestern United States gave Arizona some of its highest temperatures in the state’s history [80]. Further, pollinators in montane environments may be especially susceptible to changing climate; competition between pollinators may become more prevalent with increased elevation if the range expansion of lower elevation pollinators encroaches on the space typically reserved for the higher-elevation pollinator groups, such as bumblebees, non-syrphid flies, or cold-adapted butterflies [4,81]. These higher-elevation insect pollinators often have narrow niches restricted to upper altitudes with cooler temperatures and low seasonal temperature variation [81,82,83]. Unfortunately, areas supporting these conditions are expected to shrink disproportionately under future climate scenarios [84], potentially causing certain pollinator taxa to reduce their ranges to remain within optimal habitat [81,85]. This may impact the stability of important generalist pollinators at higher elevation environments, ultimately leading to an increased risk of community collapse.

Conservation management of pollinator species inhabiting the local mountain gradient of the San Francisco Peaks should focus on preserving and understanding the limitations of the most strongly interactive species identified in this study [23,46]. To accurately pinpoint factors that may limit these species with future climate change, their host-plant relationships and overall distribution should be studied on grander spatial scales. The response of various pollinator groups to changing climate will depend on life history traits such as sociality, body size, or nesting requirements [16], but factors that affect these species may be difficult to identify if focusing only on local studies [46]. The future development of more intensive plant–pollinator monitoring programs and pollinator inventory studies along the San Francisco Peaks will help create a more robust data set of plant–pollinator interactions in this area. Identifying the most important pollinator taxa and plant–pollinator associations could help outline steps for protecting and ensuring population health of the pollinators most responsible for network stability in this unique montane environment.

## Figures and Tables

**Table 1:** Dates and sites accessed for each sampling period. PP = ponderosa pine (~2200–2500 m), MC = mixed conifer (~2550–2700 m), SF = spruce fir (~2750–3100 m). Weather prohibited access to one site at PP in 2018, and two sites at MC in 2017. Values in each column represent site numbers. Site localities are displayed in Table S1.

Year	Sampling Dates	PP Sites Visited	MC Sites Visited	SF Sites Visited
2017	25–27 July	2, 4, 5, 6, 7, 8	3, 4, 5, 6	1, 2, 3, 4, 5, 6
2017	1–3 August	2, 4, 5, 6, 7, 8	1, 2, 3, 4, 5, 6	1, 2, 3, 4, 5, 6
2017	14–18 August	2, 4, 5, 6, 7, 8	1, 2, 3, 4, 5, 6	1, 2, 3, 4, 5, 6
2017	25–31 August	2, 4, 5, 6, 7, 8	1, 2, 3, 4, 5, 6	1, 2, 3, 4, 5, 6
2018	16–19 July	2, 4, 5, 6, 7, 8	1, 2, 3, 4, 5, 6	1, 2, 3, 4, 5, 6
2018	30 July–1 August	2, 4, 6, 7, 8	1, 2, 3, 4, 5, 6	1, 2, 3, 4, 5, 6
2018	6–8 August	2, 4, 5, 6, 7, 8	1, 2, 3, 4, 5, 6	1, 2, 3, 4, 5, 6
2018	13–15 August	2, 4, 5, 6, 7, 8	1, 2, 3, 4, 5, 6	1, 2, 3, 4, 5, 6
2018	26–30 August	2, 4, 5, 6, 7, 8	1, 2, 3, 4, 5, 6	1, 2, 3, 4, 5, 6
		52 Total	51 Total	53 Total

**Table 2:** Number of individuals and species collected during the study, reported by order.

	Total Individuals	Number of Species
Bees	384 (23% of total)	59 (38% of total)
Flies	976 (57% of total)	64 (41% of total)
Butterflies/Moths	343 (20% of total)	32 (21% of total)

**Table 3:** Number of plant and pollinator species and plant–pollinator interactions by life zone. Values are also reported by order. PP = ponderosa pine, MC = mixed conifer, SF = spruce fir.

Life Zone.	Pollinator Species	Host Plant Species	Unique Interactions	Species by Group	Interactions by Group
PP (~2200–2500 m)	73	27	163	Bees = 29 (40%) Flies = 35 (48%) Butterflies/moths = 9 (12%)	Bees = 52 (32%) Flies = 85 (52%) Butterflies/moths = 26 (16%)
MC (~2550–2700 m)	102	32	271	Bees = 34 (33%) Flies = 42 (41%) Butterflies/moths = 25 (23%)	Bees = 80 (30%) Flies = 131 (48%) Butterflies/moths = 60 (22%)
SF (~2750–3100 m)	72	20	289	Bees = 30 (41%) Flies = 24 (33%) Butterflies/moths = 18 (25%)	Bees = 51 (31%) Flies = 72 (38%) Butterflies/moths = 58 (31%)

**Table 4:** Core generalist species for each life zone (Gc values > 1.0). The number of pollinator individuals at each life zone was 340, 734, and 629 for ponderosa pine, mixed conifer, and spruce fir, respectively. The number of unique interactions at each life zone was 163, 271, and 189 for ponderosa pine, mixed conifer, and spruce fir, respectively. At each life zone, two species with the same # of interactions may receive different Gc values if there is a difference in the number of interactions when averaged across the six sample sites.

Ponderosa Pine (~2200–2500 m)				
Species	Group	Gc	Number of Individuals	% of Interactions
*Anastoechus melanohalteralis*	Fly	1.484	11 (3.2%)	3.0% (5 plants)
*Apis mellifera*	Bee	1.911	25 (7.4%)	3.6% (6 plants)
*Ashmeadiella cactorum*	Bee	1.063	13 (3.8%)	2.5% (4 plants)
*Exoprosopa rostrifera*	Fly	1.911	27 (7.9%)	2.5% (4 plants)
*Geron 001*	Fly	1.911	25 (7.4%)	3.0% (5 plants)
*Hemipenthes sinuosa*	Fly	1.487	11 (3.2%)	3.0% (5 plants)
*Melissodes confusus*	Bee	1.487	6 (1.8%)	3.6% (6 plants)
*Fannia 001*	Fly	1.897	12 (3.5%)	3.0% (5 plants)
*Muscoidea 001*	Fly	1.063	12 (3.5%)	3.0% (5 plants)
*Oarisma garita*	Butterfly	1.487	9 (2.6%)	3.6% (6 plants)
*Phthiria 003*	Fly	1.488	21 (6.2%)	1.8% (3 plants)
*Plebejus melissa*	Bee	4.032	17 (5.0%)	5.5% (9 plants)
*Ptilodexia 002*	Fly	3.183	21 (6.2%)	6.1% (10 plants)
Generalists comprise:			61.7% of total individuals	44.7% of total interactions
**Mixed Conifer (~2550–2700 m)**				
**Species**	**Group**	**Gc**	**Number of Individuals**	**% of Interactions**
*Adejeania vexatrix*	Fly	1.848	39 (5.2%)	2.6% (7 plants)
*Bombus huntii*	Bee	1.306	20 (2.7%)	2.2% (6 plants)
*Dialictus sp M2*	Bee	1.848	21(2.8%)	3.0% (8 plants)
*Geron 001*	Fly	1.306	15 (2.0%)	2.6% (7 plants)
*Hemipenthes sinuosa*	Fly	1.035	25 (3.4%)	2.2% (6 plants)
*Hylaeus cookii*	Bee	1.306	12 (1.6%)	1.8% (5 plants)
*Fannia 001*	Fly	1.014	54 (7.3%)	4.1% (11 plants)
*Muscoidea 001*	Fly	1.035	16 (2.2%)	2.2% (6 plants)
*Oarisma garita*	Butterfly	2.389	30 (4.0%)	3.3% (9 plants)
*Peleteria (Sphyrimyia) 002*	Fly	1.577	19 (2.6%)	2.6% (7 plants)
*Plebejus melissa*	Butterfly	1.577	14 (1.9%0	3.0% (8 plants)
*Polydryas arachne*	Butterfly	1.306	23 (3.1%)	2.6% (7 plants)
*Protandrena (Heterosarus) 001*	Bee	1.035	12 (1.6%)	1.8% (5 plants)
*Ptilodexia 002*	Fly	5.639	120 (16.2%)	6.3% (17 plants)
*Villa 001*	Fly	1.848	22 (3.0%)	2.6% (7 plants)
Generalists comprise:			56.6% of total individuals	42.9% of total interactions
**Spruce Fir (~2750–3100 m)**				
**Species**	**Group**	**Gc**	**Number of Individuals**	**% of Interactions**
*Anthophora terminalis*	Bee	1.203	20 (3.2%)	3.2% (6 plants)
*Fannia 001*	Fly	5.663	212 (33.7%)	6.9% (13 plants)
*Oarisma garita*	Butterfly	3.433	60 (9.5%)	5.3% (10 plants)
*Plebjeus melissa*	Butterfly	2.746	29 (4.6%)	5.8% (11 plants)
*Ptilodexia 002*	Fly	1.203	28 (4.5%)	2.6% (5 plants)
*Villa 001*	Fly	1.889	49 (7.8%)	4.2% (8 plants)
Generalists comprise:			63.3% of total individuals	28.0% of total interactions

**Table 5:** Effect on nestedness (nestedness based on overlap and decreasing fill, NODF) at the spruce fir life zone with the removal of the three most generalized pollinator species (individually and combined).

Species	Group	Gc Value	Nested Rank	New Nestedness Value	% Change in Nestedness from Original
*Fannia 001*	Fly	5.664	1	20.41	−17.06%
*Plebejus melissa*	Butterfly	2.746	2	23.57	−4.23%
*Oarisma garita*	Butterfly	3.432	3	22.17	−9.91%
All 3		n/a	n/a	15.56	−36.77%

**Table 6:** Effect on nestedness (nestedness based on overlap and decreasing fill, NODF) at the spruce fir life zone with the removal of three more specialized pollinator species (individually and combined).

Species	Group	Gc Value	Nested Rank	New Nestedness Value	% Change in Nestedness from Original
*Hylaeus cookii*	Bee	0.515	11	23.93	−2.7%%
*Thorybes pylades*	Butterfly	−0.172	28	26.96	+9.58%
*Bombus insularis*	Bee	−0.515	52	24.62	+0.0%
All 3		n/a	n/a	26.41	+7.31%

## Data Availability

Data available within Supplementary Materials, labeled as Table S4.

## Supplementary Materials

The following are available online at https://www.mdpi.com/article/10.3390/insects12121060/s1. Table S1: GPS coordinates and elevation (in meters) of 18 study site localities along the gradient of the San Francisco Peaks; Table S2: Pollinator species present at each life zone and number of life zones at which they occur. Values of “1” indicate presence; Table S3: Plant host species present at each life zone and number of life zones at which they occur. Values of “1” indicate present and observed interacting with a pollinator. A “*” symbol represents species that were present at that life zone but not observed interacting with a pollinator, therefore, they were not used in analyses for that life zone; Table S4: Complete data set for all nine sampling periods including number of plants, flowers, buds counted per plant species per site, number of pollinator individuals per species collected per site, and specific plant–pollinator interaction recorded. Note that some plant species and counts are listed that were not included in abundance/richness analyses. This is because there were no pollinator interactions observed for that species at that site. Figure S1: Bimodal networks of pollinator species (top, orange) and plant species (bottom, green) and the mutualistic interactions between species (black lines) for each life zone, where A = ponderosa pine (~2200–2500 m), B = mixed conifer (~2550–2700 m) and C = spruce fir (~2750–3100 m). The networks show interaction data combined across all sites for a life zone, although analyses were conducted on a per-site basis. Figure S2: Checkerboard visualization of plant–pollinator interaction matrices for each life zone, where A = ponderosa pine (~2200–2500 m), B = mixed conifer (~2550–2700 m) and C = spruce fir (~2750–3100 m). The figures show interaction data combined across all sites for a life zone, although analyses were conducted on a per-site basis. Plant species are labeled on the left and pollinator species are labeled on the bottom. Darker squares represent a higher number of interactions, white means no interactions. The filled squares closer to the upper left corner represent more interactive species (and plant–pollinator associations) in the plant–pollinator community. Figure S3: Regression analysis indicating correlations between statistically obtained Gc value for each pollinator species and the number of individuals collected per species at (A) ponderosa pine, (B) mixed conifer, and (C) spruce fir. Pollinator abundance was positively correlated with Gc values at all three life zones.
