# Supplementary material for: Variation in Plant–Pollinator Network Structure along the Elevational Gradient of the San Francisco Peaks, Arizona
Source: Insects. 2021 Nov 26;12(12):1060. doi: 10.3390/insects12121060 (PMC8704280; doi:10.3390/insects12121060)
Supplement: Supplementary file 1 [file insects-12-01060-s001.zip › Supp Figures and Tables/Table S1 Site Localities.pdf]

Supplemental Material 1: Locality of 18 Site Locations across the gradient

**Elevation**	**Site**	**Latitude**	**Longitude**
Ponderosa pine	2	35.4163	-111.6714
Ponderosa pine	4	35.4270	-111.6963
Ponderosa pine	5	35.3539	-111.7306
Ponderosa pine	6	35.3889	-111.7251
Ponderosa pine	7	35.3539	-111.7233
Ponderosa pine	8	35.3879	-111.6869
Mixed conifer	1	35.3285	-111.7380
Mixed conifer	2	35.3539	-111.7306
Mixed conifer	3	35.3290	-111.7390
Mixed conifer	4	35.3543	-111.7320
Mixed conifer	5	35.3803	-111.6858
Mixed conifer	6	35.3757	-111.7321
Spruce Fir	1	35.3585	-111.7080
Spruce Fir	2	35.3387	-111.6511
Spruce Fir	3	35.3322	-111.6561
Spruce Fir	4	35.3602	-111.7189
Spruce Fir	5	35.3589	-111.7181
Spruce Fir	6	35.3568	-111.7173
