# Supplementary figures and images for: Variation in Plant–Pollinator Network Structure along the Elevational Gradient of the San Francisco Peaks, Arizona
Source: Insects. 2021 Nov 26;12(12):1060. doi: 10.3390/insects12121060 (PMC8704280; doi:10.3390/insects12121060)

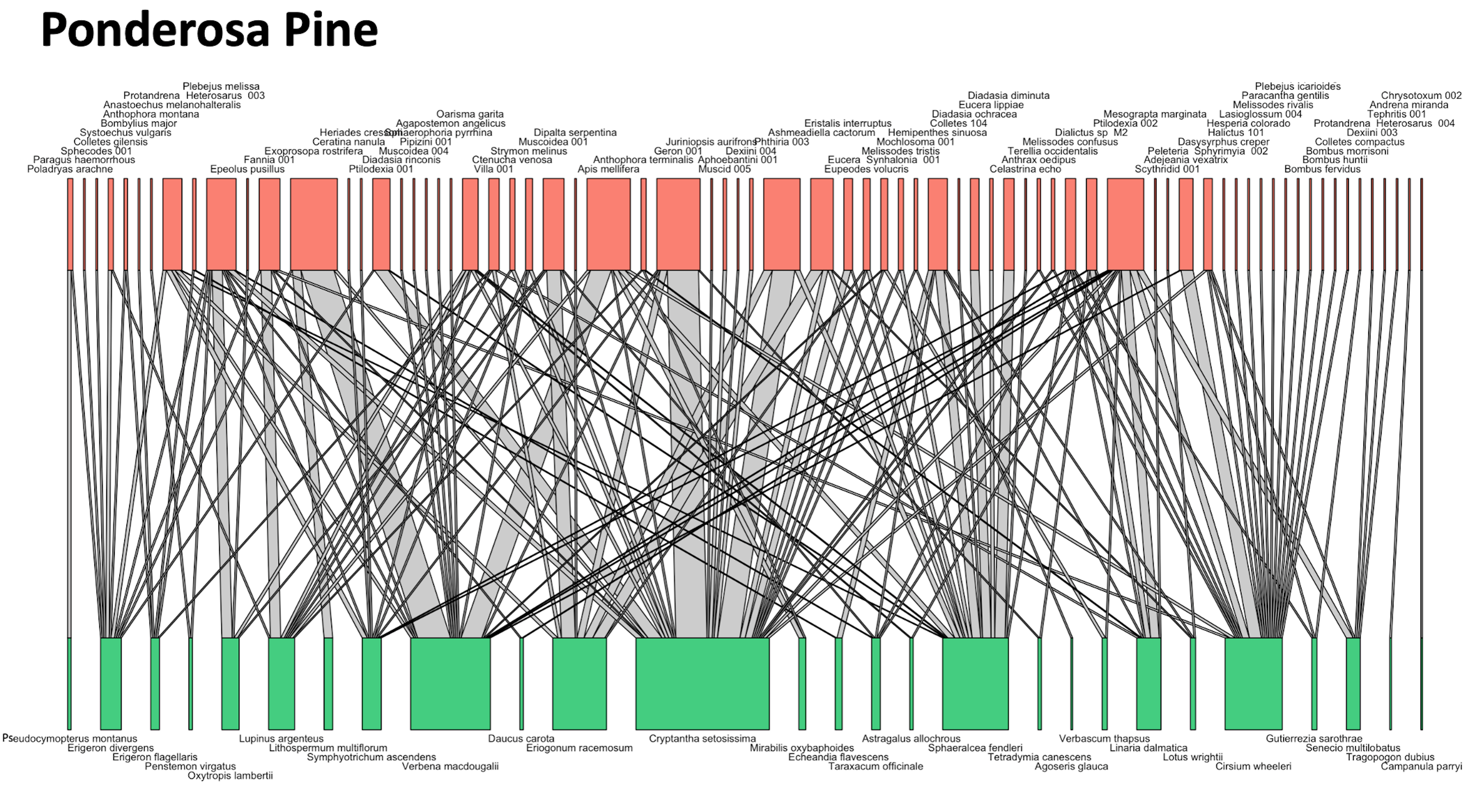

Supplement: Supplementary file 1 [file insects-12-01060-s001.zip › Supp Figures and Tables/Figure S1a.png]

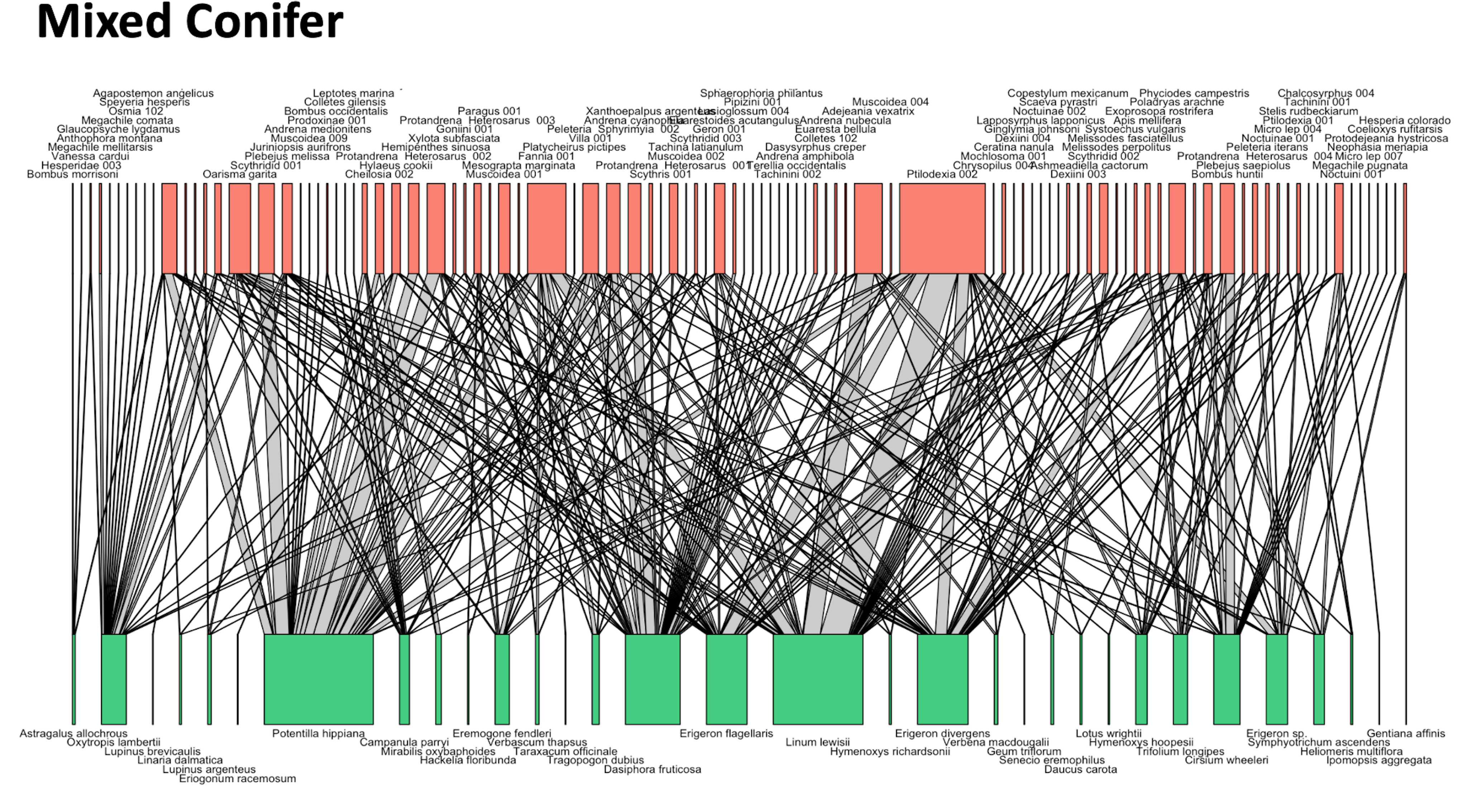

Supplement: Supplementary file 1 [file insects-12-01060-s001.zip › Supp Figures and Tables/Figure S1b.png]

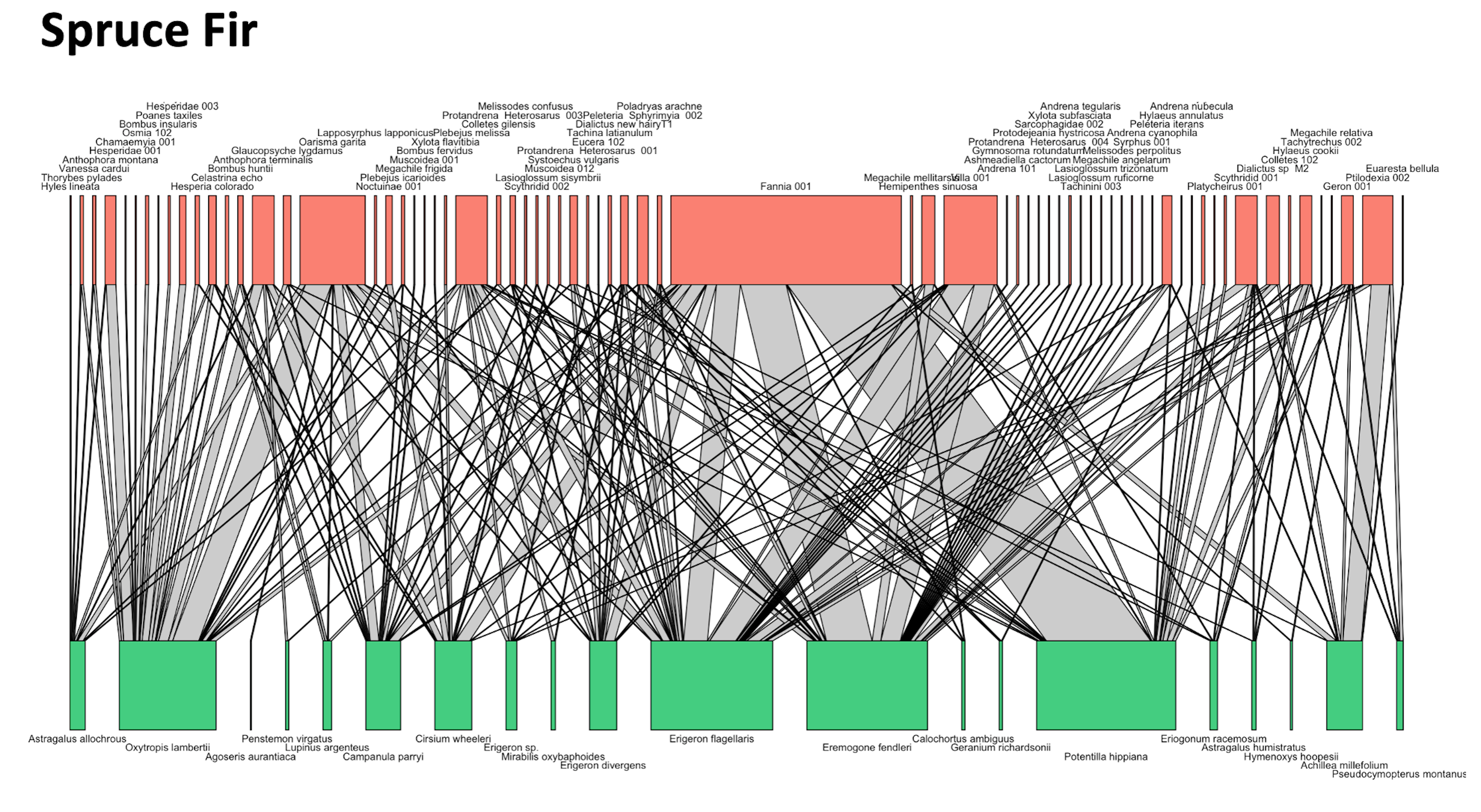

Supplement: Supplementary file 1 [file insects-12-01060-s001.zip › Supp Figures and Tables/Figure S1c.png]

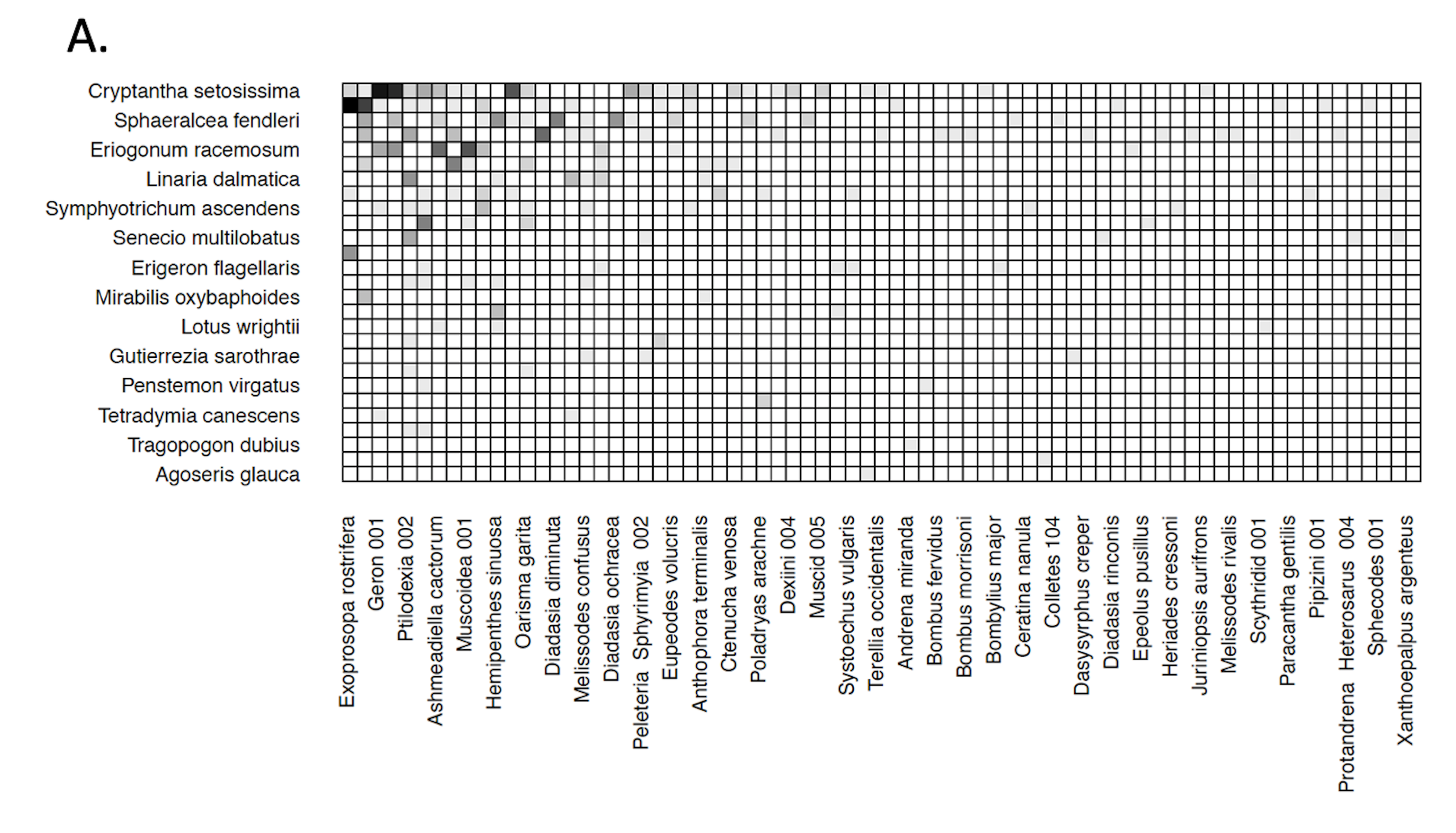

Supplement: Supplementary file 1 [file insects-12-01060-s001.zip › Supp Figures and Tables/Figure S2a.png]

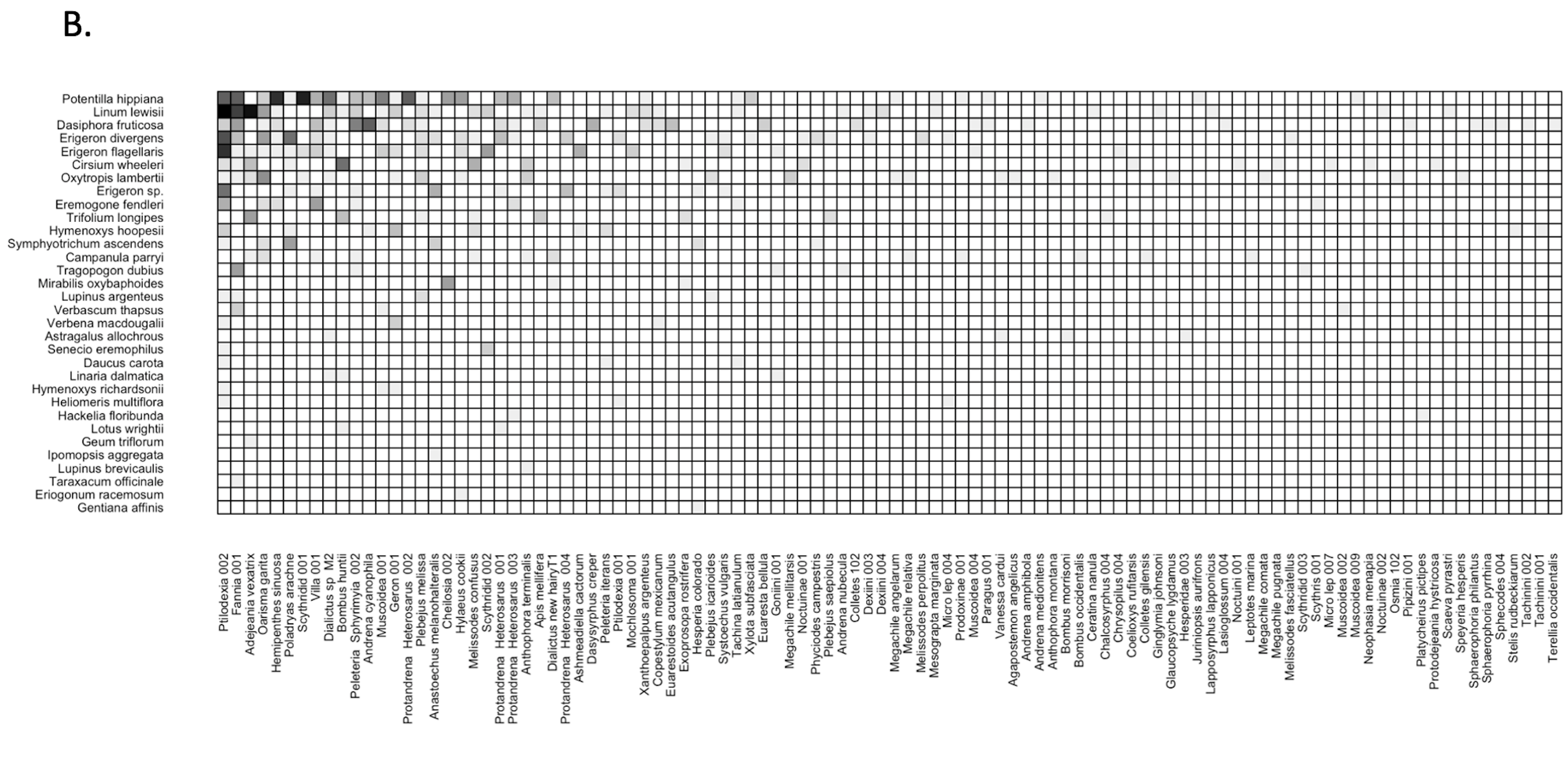

Supplement: Supplementary file 1 [file insects-12-01060-s001.zip › Supp Figures and Tables/Figure S2b.png]

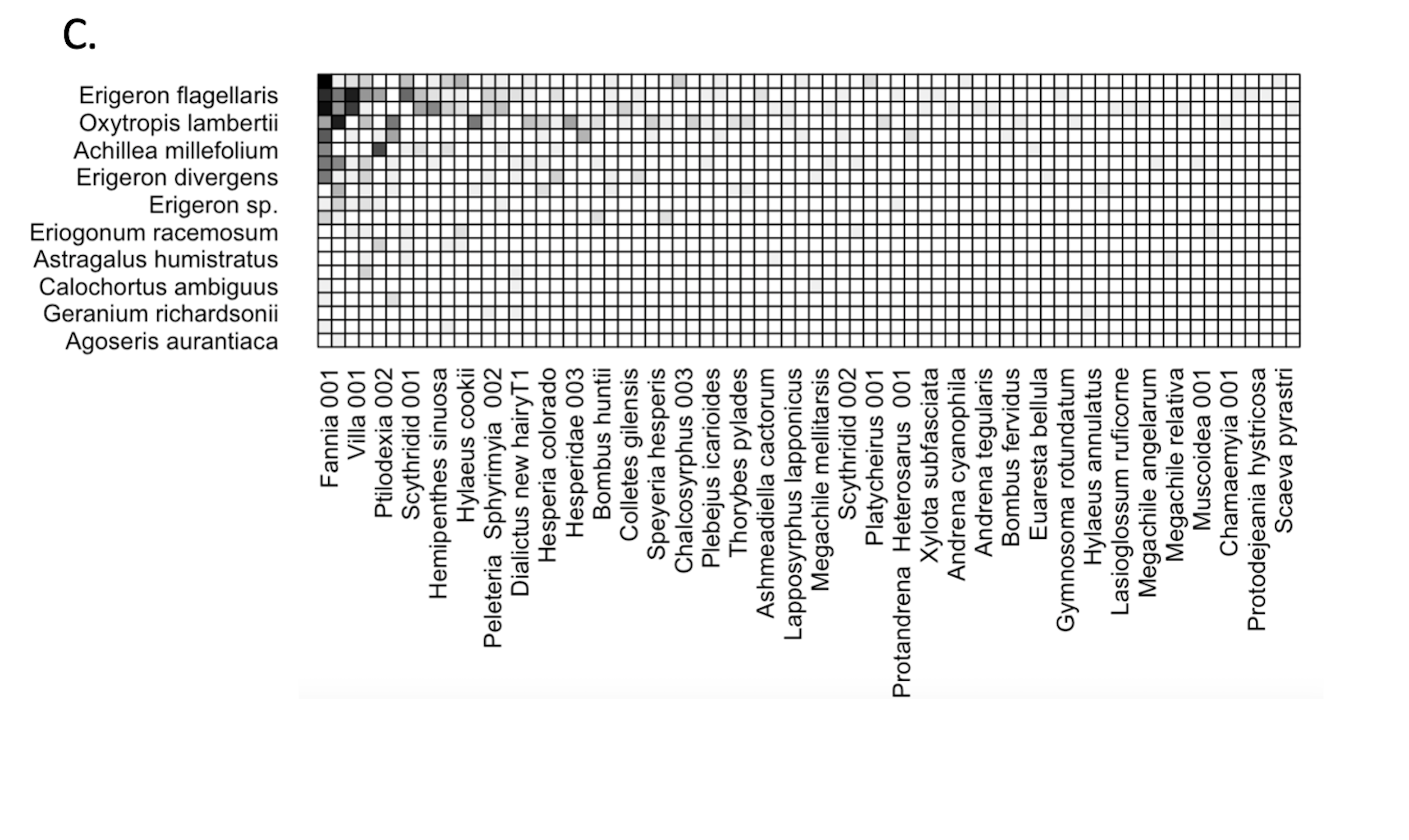

Supplement: Supplementary file 1 [file insects-12-01060-s001.zip › Supp Figures and Tables/Figure S2c.png]

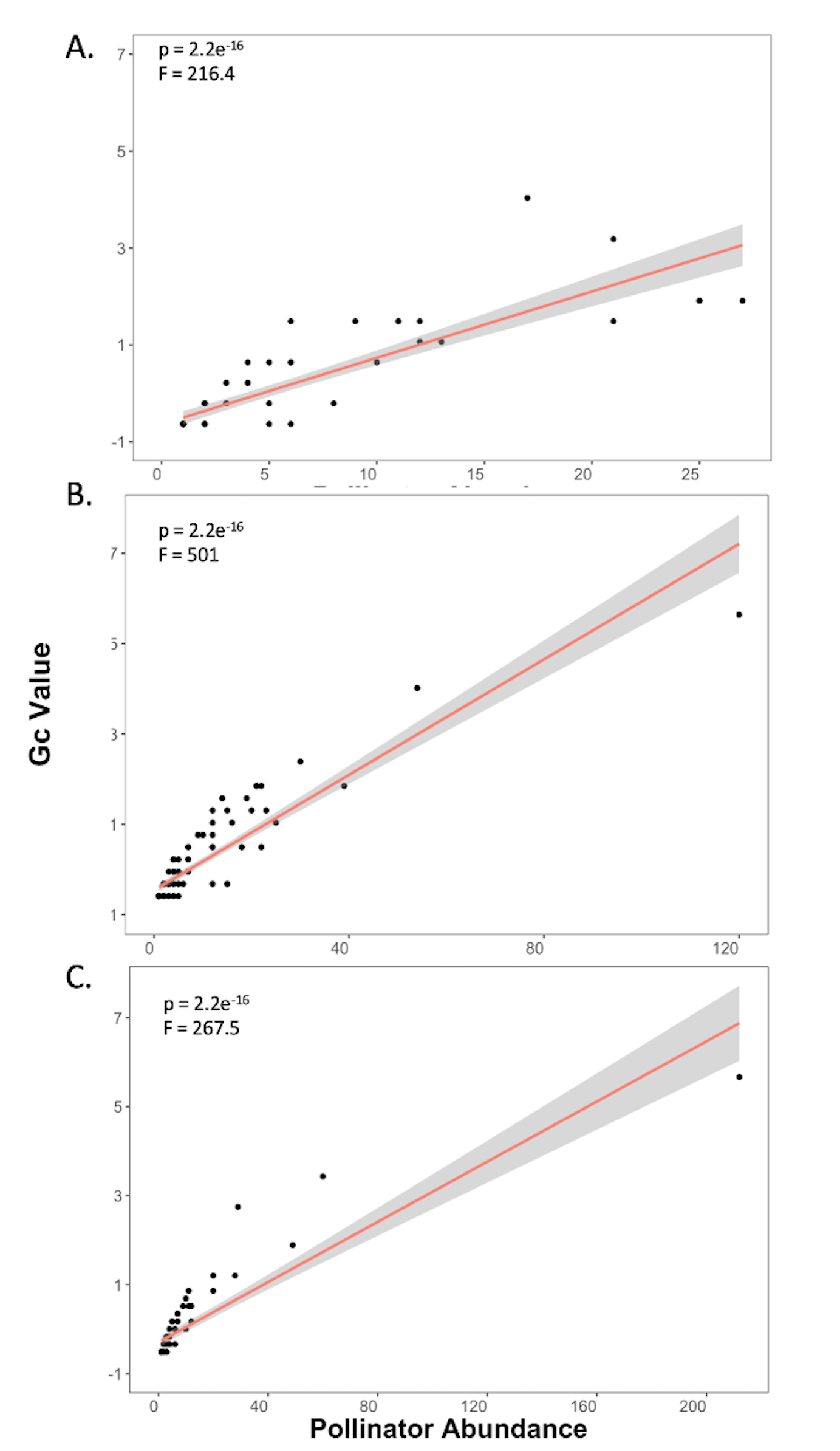

Supplement: Supplementary file 1 [file insects-12-01060-s001.zip › Supp Figures and Tables/Figure S3.png]
